# Contribution of the leaf and silique photosynthesis to the seeds yield and quality of oilseed rape (*Brassica napus* L.) in reproductive stage

**DOI:** 10.1038/s41598-023-31872-6

**Published:** 2023-03-23

**Authors:** Chunli Wang, Jianli Yang, Wenjie Chen, Xiaoguang Zhao, Zhouli Wang

**Affiliations:** Hybrid Rapeseed Research Center of Shaanxi Province, Yangling, 712100 Shaanxi China

**Keywords:** Plant physiology, C3 photosynthesis

## Abstract

Influences of photosynthesis of leaf and silique on seeds yield and quality of oilseed rape (*Brassica napus* L.) were explored in this study. A field comparing experiment with several rapeseed varieties was conducted and the results showed, that the leaf area index (LAI), silique surface area index (SAI), siliques number per plant, and biological yield were statistically classified as the first principal factors which greatly influenced seeds yield, the leaf net photosynthetic rate (*P*_n_) and silique *P*_n_ were the second principal factors; the stomatal conductance (*G*_s_) and chlorophyll *a* (Chl *a*) content were the first principal factors which influenced leaf *P*_n_ and silique *P*_n_. A shading experiment was conducted and the results showed that, under treatments of the ZH1, ZH2, and ZH3 (shading rapeseed plants during flowering stage, during time from initial flowering until seeds ripening, and during time from flowering ending until seeds ripening, respectively), respectively the seeds yield per plant was reduced by 34.6%, 84.3%, and 86.1%, the seed protein content was significantly increased. The treatment ZH1 Not, but the ZH2 and ZH3 caused significant decrease in both seed oil content and oleic acid (C18:1) content in seed oil, and the contents of linoleic acid (C18:2), linolenic acid (C18:3) in oil were significantly increased, gene expression of the ACCase (Acetyl-CoA carboxylase), FAD2 (fatty acid desaturase), and FAD3 (ω-3 fatty acid dehydrogenase) in green seeds was restrained/changed. Thus the LAI, SAI, siliques number per plant, biological yield per plant, leaf *P*_n_, silique *P*_n_, and the *G*_s_, Chl *a* content of leaf and silique formed an indexes system to be used in screening rapeseed variety with higher light efficiency and seeds yield; the silique photosynthesis inhibition and the photosynthates deficiency in rapeseed plant after flowering stage predominately influenced seeds yield and quality.

## Introduction

The oilseed rape (*Brassica napus* L.) is widely planted all over the world, seeds oil of the crop is edible and healthy, also it is higher quality lubricating oil and is largely consumed in industry, and the oil is potential energy material in synthesizing biodiesel, consumption of the biodiesel rather than the low sulphur diesel would save energy and alleviate potential global warming^1,2^. The demands for the rapeseed oil increased rapidly in recent years, to deal with the crisis of food and energy shortage, relevant researches are important and are urgently needed in screening oilseed rape variety with high seeds yield and quality, and in advancing cultivation techniques.

Photosynthetic capacity of the C3 plant rapeseed during reproductive stage greatly influenced seeds yield formation and seed oil quality, the production and accumulation of dry matters in the plant at the stage was linearly related to siliques number per plant and seeds density per silique^3^. Defoliation treatment led inhibition of siliques formation and growth in *Brassica* species, leaf photosynthesis accelerated seeds development^4^. To rapeseed plant after flowering the siliques rapidly developed and the leaves area rapidly reduced, eventually the surface area of siliques exceeded or equaled to that of leaves^5,6^; the fully developing siliques layer intercepted and absorbed about 80% of solar radiation, the CO_2_ amount fixed by siliques exceeded that fixed by leaves in two weeks post anthesis, about 80–95% of total photosynthetic assimilates in silique was generated by the silique itself photosynthesis^7^. The shading-silique treatment application on *Brassica napus* L. during plant reproductive stage led decrease of 60% seeds yield, the seed oil content was decreased by 44.7%, the fatty acids proportion of seed oil was changed^8^. According to relationships between the photosynthetic capacity of rapeseed plant, assimilates accumulation in seed and the seeds yield, a statistical model was derived to assess seeds yield of rapeseed variety^9^. It is apparent that the seeds yield and quality were crucially influenced by photosynthesis of rapeseed plant during reproductive growth stage, in plant the assimilates was mainly produced and supplied by leaves photosynthesis in flowering stage, and by siliques layer photosynthesis after flowering; the mechanism on the leaf and silique photosynthesis influencing seeds yield and quality demands a systematical further exploration.

The glucose is a photosynthetic product and carbon source in biosynthesizing fatty acids. It was converted into hexose through the glycolysis metabolic pathway, and then the hexose was oxidized and translated into acetyl-CoA. The acetyl-CoA was a precursor to be used in synthesizing saturated and unsaturated long chain fatty acids, under catalytic action of key enzymes such as the Acetyl-CoA carboxylase (ACCase), fatty acid desaturase (FAD2), ω-3 fatty acid dehydrogenase (FAD3, FAD7 and FAD8), and fatty acid lengthening enzyme (FAE)^10–12^. The activity of chloroplast ACCase was enhanced in culture of the *Phaeodactylum tricornutum* under unfavorable culture conditions, the contents of neutral lipid, fat, monounsaturated fatty acids in the culture were increased^13^. The genes of fatty acid synthesis enzymes such as the *ACCase*、*BC* (Biotincarboxylase), *AhBC4* (subunit of heterogeneic ACase), *AhKASII* (ketoacyl-ACP synthase), *AhSAD* (stearoyl-ACP desaturase), and diacylglycerol transferases were over-expressed in peanut (*Arachis hypogaea* L.) by transgenic technology, oil body protein genes of the *AhDGAT1*, *AhDGAT2*, *AhOle1*, *AhOle2* and *AhOle3* were over-expressed also, then the seed oil content and seed weight were increased significantly, the contents of stearic acid, oleic acid and linoleic acid in seed oil were altered markedly^14^. That’s to say, the synthesis and accumulation of fatty acids in plant seed or algae culture were affected by gene expression level and activity of the key enzymes in fatty acids synthesis pathway.

The yields formation was mainly dominated by accumulation of photosynthetic products in oilseed rape. In this study, a field experiment with several rapeseed varieties was conducted, correlative relationship between the seeds yield and the leaf photosynthesis, silique photosynthesis, siliques number per plant, seeds number per silique, 1000-seed weight, and biological yield was studied, several phenotypic and physiologic indexes were selected for screening rapeseed variety with higher seeds yield and quality; furthermore, a shading experiment with a rapeseed variety was conducted to quantitatively evaluate effects of the leaf and silique photosynthesis on seeds yield, seed oil content, and fatty acids synthesis. Basing on these experiments, the contribution of leaf and silique photosynthesis to seeds yield and quality, as well as the influencing mechanism were elucidated in rapeseed variety.

## Materials and methods

### Experimental site and the soil

In this study the field experiments were arranged in field of the Hybrid Rapeseed Research Center of Shaanxi Province, China. The experimental field (108° 08′ E, 34° 20′ N, 521 m above sea level) located in semi-humid warm temperate zone with continental monsoon climate. The average annual rain precipitation was about 650 mm and average annual temperature was 12.9 °C. The ground of experiment field was loess soil with organic carbon content 8.14 g kg^−1^, total nitrogen content 0.95 g kg^−1^, total phosphorus content 0.83 g kg^−1^, total potassium content 20.42 g kg^−1^, available phosphorus content 0.021 g kg^−1^, and exchangeable potassium content 0.29 g kg^−1^.

### The comparing experiment

The field comparing experiment was conducted in 2019–2021, with 11 rapeseed varieties (*Brassica napus* L.): the *Feng-You*-737 (F-737), *You-Yan*-9 (Y-9), *Rong-You*-8 (R-8), *Rong-You*-11 (R-11), *Qin-You*-10 (Q-10), *Qin-You*-7 (Q-7), *Qin-You*-88 (Q-88), *Qin-You*-33 (Q-33), *De-Zhong-You*-1 (D-1), *Hua-You*-2 (H-2), and *Feng-You*-679 (F-679). All these rapeseed varieties were winter ecotype or semi-winter ecotype hybrid, they were suitable for planting in the Yangtze River valley and the Huang-Huai River region in China. Plant height of the tested rapeseed varieties was about 1.5 m. Phenological periods of the rapeseed varieties were uniform.

The 11 rapeseed varieties were sowed at 25th September, the initial flowering date and ending flowering date was on 23th March and 18th April around, respectively. The harvest date was about at 28th May. These oilseed varieties mainly bloomed in April and seed mainly developed in May in 2019–2021, respectively the maximum mean air temperature, minimum mean air temperature, sunny days and rainy days was 21/18°, 7/8 °C, 6/5 days, and 5/13 days in April 2020/2021, and was 28/27 °C, 14/14 °C, 5/6 days, and 7/6 days in May 2020/2021. The maximum mean air temperature was lower, there was more rainy days in April–May 2021, compared to that in April–May 2020.

Every rapeseed variety was repeatedly sowed in three plots, with 0.4 m of row spacing and 0.12 m of plant spacing, plants density was about 200,000 plants·hm^−2^. The plots were randomly arranged with 0.8 m gap between two plots. Area in every plot was 3.0 m × 4.0 m; the sampling area was 2.5 m × 2.4 m and located in middle of every plot. Normal farming managements were applied in the experiment. Before sowing fertilizers were applied in the plots according to levels of ammonium bi-phosphate (NH4H2PO4) 187.5 kg ha^−1^, urea 150 kg ha^−1^, and borate fertilizer (11.3% of boron content) 7.5 kg ha^−1^. All these plots were irrigated in December.

### The shading experiment

The shading experiment was conducted in time from September 2020 to July 2021, using the “Q-7” variety as material; the “Q-7” variety had higher adaptability and higher seeds yield and was widely planted, which was frequently designed as a Control in the national regional comparative trial of rapeseed variety in China. The shading experiment including four treatments: the ZH1 treatment that rapeseed plants were shaded during flowering stage (in time from 23th March to 18th April), the ZH2 treatment that rapeseed plants were shaded in time from initial flowering until seed ripening (in time from 23th March to 28th May), the ZH3 treatment that rapeseed plants were shaded in time from flowering ending until seed ripening (in time from 18th April to 28th May), and the Control that rapeseed plants grew under nature light. Every treatment was repeated in three plots. The sowing date, harvesting date and culturing managements were same as above. The shading shed was built with steel tube frame and covered with shading net on roof, east side, south side, and west side, respectively, while keeping the north side open. The shed height was 2.5 m, field area in the shed was 3.0 m × 4.0 m (sampling area was 2.5 m × 2.4 m and located in middle of the plot). The gap between two sheds was 3.0 m. The light intensity inside/outside shed was about 150/1200 µmol m^−2^ s^−1^ at 11:30 p.m. in sunny day during flowering stage of the rape variety, and it was 250/1500 µmol m^−2^ s^−1^ during silique development stage.

### Measurement of photosynthetic parameters of leaf and silique

The net photosynthetic rate (*P*_n_), stomatal conductance (*G*_s_), and transpiration rate (*T*_r_) were measured by a portable photosynthesis system (Li-6400, USA) under natural temperature. The first short stalk leaf on main stem, or silique at middle position of main inflorescence of rapeseed plant was selected and measured (the leaf and silique were attached on plant), respectively. The measuring condition was designed as an open-circuit gas channel system, 500 mol s^−1^ air velocity, and 400 mol·mol^−1^ CO_2_ concentration. The measurement was conducted in sunny day, the light intensity outside/inside the shading shed were set at 1200 µmol m^−2^ s^−1^/150 µmol m^−2^ s^−1^ during plants flowering period, and they were set at 1500 µmol m^−2^ s^−1^/250 μmol m^−2^ s^−1^ during silique development stage^15^. These detections were repeated on 5 plants in each plot.

### Leaf area index (LAI) and silique area index (SAI) of rapeseed variety

During full blooming stage (at 7th day after initial flowering) or seed filling stage (at 22th day after flowering ending), surface area of leaves/green siliques per plant was measured by a leaf area meter (Yaxin–1241, China). To determine the siliques surface area per plant, fifty green siliques were randomly picked from different branches on a plant, every silique hull was split along crack line and flattened, then surface area of the fifty siliques hull was measured; meanwhile, fresh weights of the leaves and siliques were measured, respectively. According to fresh weight of total siliques on a plant, the siliques surface area per plant was calculated. These items were repeatedly measured on 3 plants in a plot, these data were averaged. Then fresh weight of total leaves or total siliques of 10 plants in a plot was measured (including above plants measured by the Yaxin–1241 leaf area meter), and the sample area was investigated. Basing on these results the LAI or the SAI was concluded.

### Contents of photosynthetic pigments and photosynthetic enzyme protein in leaf and silique hull

The contents of chlorophyll *a* (Chl *a*) and chlorophyll *b* (Chl* b*) in leaf and silique hull were determined according to the Acetone Colorimetric Method (Gao, 2006), the content of the Ribulose-1,5-bisphosphate carboxylase/oxygenase (RuBisCO, EC 4.1.1.39) protein was measured by an ELISA test kit.

### Yield indexes

At silique ripening stage, a total of three plants in a plot were randomly selected, the siliques number per plant was measured; a total of 50 siliques were picked from upper, middle, and lower branches of these plants, the seeds number per silique was investigated. Plants in 1.2 × 0.8 m^2^ area in each plot (a total of 20 plants) were harvested and invested for the 1000-seed weight, biological yield, and seeds yield.

### Seed quality and fatty acids content in seed oil

The oil content in seed was measured by the NMR method (NMR Analyzer, mq20, BRUKER, Germany); the seed protein content was measured by the Near–Infrared Spectroscopy Method (Fourier transform NIR spectrometer, Matrix-I, BRUKER, Germany); the glucosinolate content in seed was measured by the Liquid Chromatography Method (UPLC H-class, Waters, America); the fatty acids content in seed oil was measured by the Gas Chromatography Method (6890N GC, Aglient, America); these were repeated for three times.

### Gene expression of the enzymes related to fatty acids biosynthesis

In seeds filling stage (at the 25th day post anthesis), green seeds were collected to extract RNA. Then, gene expression level of the acetyl coenzyme A carboxylase (*ACCase*), oleic acid desaturase (*FAD2*), and omega-3 fatty acid desaturase (*FAD3*) were detected by the Real-Time Fluorescence Quantitative PCR (RT-q PCR) technique with the RT-q PCR instrument (ABI 7500, the Applied Biosystems, America), with three repeats. The gene expression levels were analyzed according to the 2^−ΔΔCt^ method^16,17^. The RT-q PCR primers of target genes were designed and shown in the Table 1.

**Table 1:** The RT-q PCR primers of gene of the enzymes related to fatty acid biosynthesis.

Gene	Primer sequence (5′–3′)
*FAD2*	Forward: GCTGGCGTCCTCTCCGTATGTTA
	Reverse: CGTGCGTGTCCGTGATGTTATGA
*FAD3*	Forward: TTCCCACAAATCCCTCACTATCA
	Reverse: ACTTGCCACCAAACTTTCCACC
*FAE1*	Forward: GTCAGGCTTTAAGTGTAACAGTGCA
	Reverse: TTATTAGGACCGACCGTTTTGG
*ACCase*	Forward: AGGACTTGCCAATCTTCTAAAC
	Reverse: AGCTTCTTTCACCGTAGGACAC
*Action*	Forward: TCTTCCTCACGCTATCCTCCG
	Reverse: AGCCGTCTCCAGCTCTTGC

### Research involving plants

The authors declare that all local, national or international guidelines and legislation were adhered for the use of plants in this study. The 11 rape varieties used in the study were registered and allowed for sale in market in China. The Q-7, Q-88, and Q-33 were cultivated and supplied by the Hybrid Rapeseed Research Center of Shaanxi Province, the F-737, Y-9, R-8, R-11, Q-10, D-1, H-2, and F-679 were purchased in seed market in China.

### Statistical analysis

The experiment data was statistically analyzed by the Excel 2010 software and the DPS V7.55 analysis software. The principal influencing factors were analyzed according to the Principal Factor Regression Method; and significant difference was elucidated according to the Duncan’s Method.

## Results

### Relationships between leaf photosynthesis, silique photosynthesis and seeds yield

According to results of the comparing experiment conducted in 2019–2020 with a total of 11 rapeseed varieties (Table 2), the LAI decreased from 4.70 to 2.07, the SAI decreased from 3.50 to 1.33, the siliques number per plant generally decreased from 277 to 170, the aboveground biological yield decreased from 9.60 to 4.65 t ha^−1^, and the seeds yield decreased from 4.51 to 1.96 t ha^−1^. In the experiment conducted in 2020–2021 with 11 rapeseed varieties, the LAI gradually decreased from 5.32 to 3.45, the SAI generally decreased from 3.64 to 2.30, the siliques number per plant decreased from 270 to 189, the aboveground biological yield viewed significant decreasing trend, and the seeds yield decreased from 5.18 to 2.95 t ha^−1^. Obviously, the LAI, SAI, siliques number per plant, aboveground biological yield, and seeds yield showed a similar change trend in different rapeseed varieties.

**Table 2:** The photosynthetic indexes and yields of different rapeseed varieties in the comparing experiment.

Growing season	Variety	Leaf *P*_n_ (μmolCO_2_ m^−2^ s^−1^)	Leaf LAI	Silique *P*_n_ (μmolCO_2_ m^−2^ s^−1^)	Silique SAI	Seeds number per silique	1000-seed weight (g)	Siliques number per plant	Aboveground biological yield (t ha^−1^)	Seeds yield per plant (t ha^−1^)
2019–2020	F-737	26.3 de	4.70 a	9.9 cd	3.50 a	24.6 cd	4.38 d	277 ab	9.60 a	4.51 a
	Y-9	23.3 f	4.86 a	11.1 bc	2.44 bc	28.4 abc	4.03 de	258 b	9.37 a	3.85 ab
	R-8	28.3 abc	4.56 a	12.1 ab	2.07 cd	28.7 abc	4.03 de	283 a	9.30 a	3.51 bc
	R-11	26.9 d	3.49 c	12.1 ab	2.56 b	26.2 bcd	4.33 d	215 cd	8.67 a	3.47 bc
	Q-10	28.7 a	3.95 b	8.8 de	2.78 b	28.2 abc	3.47 e	226 c	7.97 a	3.29 bc
	Q-7	27.8 bc	3.49 c	13.4 a	2.49 bc	33.6 a	3.52 e	201 de	8.02 a	3.20 bc
	Q-88	28.5 ab	4.57 a	10.7 bc	1.94 d	26.2 bcd	5.75 a	228 c	8.75 a	3.13 bcd
	Q-33	27.7 c	3.52 c	11.1 bc	1.62 de	31.6 ab	4.55 cd	195 de	7.47 ab	2.84 cd
	D-1	26.2 de	2.56 d	13.8 a	1.99 d	21.2 d	5.24 abc	191 def	6.53 ab	2.82 cd
	H-2	26.8 d	3.17 c	7.4 e	1.43 e	28.0 abc	5.45 ab	181 ef	7.69 ab	2.31 de
	F-679	25.9 e	2.07 e	11.1 bc	1.33 e	24.4 cd	4.80 bcd	170 f	4.65 b	1.96 e
2020–2021	Q-1	17.5 cde	5.32 ab	8.9 cd	3.64 b	31.1 a	4.65 c	270 a	14.53 a	4.90a
	R-11	17.1 def	5.67 a	10.7 a	4.01 a	24.7 e	3.94 f	269 a	14.24 a	4.80 ab
	R-1	16.2 f	3.80 bc	6.2 f	3.29 c	27.2 cd	4.15 ef	218 de	12.46 b	4.35 bc
	Q-33	17.6 cd	4.02 abc	9.3 bc	3.08 cd	28.7bc	4.67 c	223 cde	12.29 b	4.14 cd
	Q-7	18.0 cd	4.31 abc	10.0 ab	2.96 d	29.3 b	3.93 f	233 cd	11.64bc	3.95 cde
	S-16	18.6 bc	3.50 bc	9.8 b	3.25 c	27.1 cd	4.88 bc	257 ab	11.54 bcd	3.77 de
	S-15	16.4 ef	4.31 abc	9.7 b	2.44 fg	26.7 d	4.67 c	247 abc	11.87 b	3.56 ef
	S-803	20.9 a	3.45 c	8.4 de	2.72 e	24.1 e	4.30 de	243 bc	10.89 bcd	3.21 fg
	S-28	15.0 g	4.07 abc	8.5 de	2.59 ef	29.0 b	5.02 b	203 ef	8.81 e	3.14 fg
	Q-10	17.0 def	3.97 abc	7.8 e	2.31 g	27.1 cd	4.40 d	175 g	9.74 de	3.08 fg
	Q-88	19.2 b	3.45 c	9.6 b	2.30 g	23.7e	5.41 a	189 fg	9.83 cde	2.95 g

LAI, leaf area index; SAI, silique surface area index; *P*_n_, net photosynthetic rate. Different lowercase letters in same column indicated significant differences (*P* < 0.05). The same below.

A regression equation was statistically calculated according to correlational relationships between the seed yield (*Y*) and the yield-related indexes (the indexes *X*_1,_ *X*_2,_ *X*_3,_ *X*_4,_ *X*_5,_ *X*_6,_ *X*_7,_ *X*_8_) of rapeseed variety, with correlation coefficient *R* = 0.9697, determination coefficient *R*^2^ = 0.9402, and *P* = 0.0001, as follows:

$$Y = - 1.0036\,+\,0.0125X_{1}\,+\,0.1673X_{2}\,+\,0.0255X_{3}\,+\,0.6322X_{4}\,+\,0.0128X_{5}\,+\,0.0195X_{6}\,+\,0.0020X_{7}\,+\,0.0790X_{8}$$

According to the statistical results, the LAI, SAI, siliques number per plant, and aboveground biological yield were classified as the first principal factors which influenced seeds yield of rapeseed variety, with 46.1% of contribution rate to the seed yield; the leaf *P*_n_ and silique *P*_n_ were classified as the second principal factors, with 21.1% of contribution rate to the seed yield; respectively the seeds number per silique, the 1000-seed weight was the third, fourth principal factor, with 13.6% of contribution rate to the seed yield. The cumulative contribution rate of the four principal factors to seeds yield was about 88.8% (Table 3). In short, in influencing seeds yield of rapeseed variety the LAI, SAI, siliques number per plant and aboveground biological yield were the first principal factors, the leaf *P*_n_ and silique *P*_n_ were the second principal factors.

**Table 3:** The principal factors which mainly influenced seeds yield of rapeseed variety.

Principal factor	Vector eigenvectors of the variable parameter								Contribution percentage (%)	Cumulative contribution percentage (%)
	Leaf *P*_n_ (*X*_1_)	Leaf LAI (*X*_2_)	Silique *P*_n_ (*X*_3_)	Silique SAI (*X*_4_)	Seeds number per silique (*X*_5_)	1000-seed weight (*X*_6_)	Siliques number per plant (*X*_7_)	Biological fresh yield (*X*_8_)		
z1	− 0.326	0.436	− 0.192	0.469	0.112	− 0.211	0.390	0.488	46.1	46.1
z2	0.487	0.181	0.518	0.001	0.343	− 0.484	0.294	− 0.156	21.1	67.2
z3	0.147	0.102	0.392	0.135	− 0.750	0.297	0.381	0.028	13.6	80.7
z4	0.169	0.434	− 0.024	− 0.314	0.402	0.691	0.200	0.064	8.1	88.8

### The principal factors which influenced net photosynthetic rate of leaf and silique

According to results of the comparing experiment conducted in 2019–2020, in different rapeseed varieties the leaf *P*_n_ decreased from 28.7 to 26.2 μmolCO_2_ m^−2^ s^−1^, correspondingly the leaf *G*_s_ decreased from 0.969 to 0.792 mol H_2_O m^−2^ s^−1^, and the leaf Chl *a* content viewed decreasing trend. In the experiment conducted in 2020–2021, in different rapeseed varieties the leaf *P*_n_ decreased from 20.9 to 16.4 μmolCO_2_ m^−2^ s^−1^, correspondingly the leaf *G*_s_ viewed decreasing trend (Table 4). In a word, the leaf *P*_n_, *G*_s_, and Chl *a* content generally showed a similar change trend in different rapeseed varieties.

**Table 4:** Photosynthetic parameters, pigments content and RuBisCO content of leaf and silique hull of different rapeseed varieties.

Tissue (growing season)	Rape variety	*G*_s_ (mol m^−2^ s^−1^)	*T*_r_ (mmol m^−2^ s^−1^)	Chl *a* (mg g^−1^)	Chl* b* (mg g^−1^)	RuBisCO (ng g^−1^)	*P*_n_ (μmol m^−2^ s^−1^)
Leaf (2019–2020)	Q-10	0.969 a	5.13 bc	1.346 a	0.372 a	211.9 c	28.7 a
	Q-88	0.935 a	4.63 d	1.252 ab	0.325 ab	176.8 f	28.5 ab
	R-8	0.947 a	5.71 a	1.081 bcd	0.292 b	176.8 f	28.3 abc
	Q-7	0.805 b	4.54 d	1.16 abc	0.318 ab	194.1 e	27.8 bc
	Q-33	1.123 a	5.19 bc	1.186 abc	0.312 ab	182.4 f	27.7 c
	R-11	0.956 a	5.53 ab	1.049 bcd	0.294 b	230.2 b	26.9 d
	H-2	0.747 b	4.84 cd	1.003 cd	0.262 bc	150.5 h	26.8 d
	F-737	0.780 b	4.85 cd	1.354 a	0.367 a	162.2 g	26.3 de
	D-1	0.792 b	5.31 ab	1.026 cd	0.266 bc	205.8 cd	26.2 de
Leaf (2020–2021)							
	S-803	0.374 a	4.02 a	0.874 de	0.251 cd	330.2 b	20.9 a
	Q-88	0.291b	3.41 b	0.887 cde	0.252 cd	209.2 f	19.2 b
	S-16	0.222 cd	2.90 d	1.037 a	0.296 a	379.4 a	18.6 bc
	Q-7	0.266 b	3.18 bc	0.856 e	0.256 bc	181.7 g	18.0 cd
	Q-33	0.275 b	3.34 b	0.93 bc	0.269 b	303.7 c	17.6 cd
	Q-1	0.271 b	3.26 b	0.845 e	0.237 de	337.6 b	17.5 cde
	R-11	0.282 b	3.17 bc	0.759 f	0.224 e	272.7 d	17.1 def
	Q-10	0.226 cd	2.42 f	0.979 b	0.286 a	151.1 h	17.0 def
	S-15	0.237 c	2.94 cd	0.838 e	0.234 e	245.6 e	16.4 ef
Silique hull (2019–2020)	D-1	0.351 a	6.03 a	0.256 a	0.085 a	249 c	13.8 a
	Q-7	0.239 b	4.34 b	0.172 d	0.054 bc	219 e	13.4 a
	R-11	0.402 a	6.64 a	0.230 b	0.085 a	266 b	12.1 ab
	R-8	0.257 b	4.53 b	0.182 cd	0.052 bc	233 d	12.1 ab
	F-679	0.350 a	6.14 a	0.181 cd	0.058 b	345 a	11.1 bc
	Q-33	0.227 b	3.98 b	0.201 c	0.059 b	225 de	11.1 bc
	Y-9	0.165 c	2.96 c	0.178 d	0.047 bcd	241 c	11.1 bc
	Q-88	0.233 b	4.06 b	0.169 d	0.052 bc	206 f	10.7 bc
	F-737	0.148 c	2.74 c	0.161 de	0.044 cd	197 g	9.9 cd
Silique hull (2020–2021)	R-11	0.203 a	4.80 ab	0.150 bc	0.040 cd	245.1 e	10.7 a
	Q-7	0.167 bc	4.32 ab	0.132 de	0.044 bc	178.2 g	10.0 ab
	S-16	0.162 bc	5.24 a	0.138 d	0.043 bc	341.1 a	9.8 b
	S-15	0.160 bc	4.00 bc	0.129 de	0.044 abc	229.9 f	9.7 b
	Q-88	0.165 bc	4.60 ab	0.154 ab	0.050 a	170.3 h	9.6 b
	Q-33	0.170 bc	3.23 c	0.129 de	0.040 cd	266.8 d	9.3 bc
	Q-1	0.147 cd	4.80 ab	0.140 cd	0.045 abc	297.8 b	8.9 cd
	S-28	0.191 ab	2.99 c	0.121 e	0.029 e	261.8 d	8.5 de
	S-803	0.160 bc	4.99 ab	0.104 f	0.036 d	287.4 c	8.4 de

*G*_s_, stomatal conductance; *T*_r_, transpiration rate; Chl *a*, chlorophyll *a*; Chl* b*, chlorophyll *b*. The same below.

According to results of the comparing experiment conducted in 2019–2020, in different rapeseed varieties the silique *P*_n_ decreased from 13.8 to 9.9 μmolCO_2_ m^−2^ s^−1^, meanwhile the *G*_s_, *T*_r_, and Chl *a*, Chl *b* content of the silique hull decreased also. In the experiment conducted in 2020–2021, in different rapeseed varieties the silique *P*_n_ decreased from 10.7 to 8.4 μmolCO_2_ m^−2^ s^−1^, also the *G*_s_ and Chl *a* content of the silique hull showed decreasing trend. (Table 4). The *G*_s_, *T*_r_ and Chl* a*, Chl *b* content of silique hull generally showed a similar change trend in different rapeseed varieties.

A regression equation was statistically calculated according to correlation relationships between the photosynthetic parameters (the *X*_1,_ *X*_2,_ *X*_3,_ *X*_4,_ *X*_5_) and the leaf *P*_n_ (*Y*) of rape variety, with *R* = 0.9822, *R*^2^ = 0.9647, and *P* = 0.0001, as follows:

$$Y = {5}.{7643}\,+\,{5}.{9518}X_{{1}}\,+\,{2}.0{933}X_{{2}}\,+\,{3}.{7513}X_{{3}}\,+\,{6}.{8358}X_{{4}}\,-\,0.00{39}X_{{5}} .$$

According to the statistical results, in influencing leaf *P*_n_ the first principal factors included the leaf *G*_s_ and Chl *a* content, with contribution rate 71.1%; the second principal factors included the leaf Chl* b*, RuBisCO content, with contribution rate 15.9%; the third principal factor was the 1eaf *T*_r_, with contribution rate 12.2%; the cumulative contribution rate of these principal factors was about 99.2% to the leaf *P*_n_ (Table 5). In brief, the *G*s, Chl *a* content were the first principal factors that affected leaf *P*_n._

**Table 5:** The principal factors which affected leaf *P*_n_.

Principal factor	Vector eigenvector of the variable parameter					Contribution percentage (%)	Cumulative contribution percentage (%)
	Leaf *G*_s_ (*X*_1_)	Leaf *T*_r_ (*X*_2_)	Chl *a* (*X*_3_)	Chl *b* (*X*_4_)	RuBisCO (*X*_5_)		
z1	0.4995	0.4528	0.4887	0.4479	− 0.3256	71.1	71.1
z2	− 0.187	− 0.2679	0.3933	0.5377	0.6704	15.9	87.0
z3	0.3395	0.5779	− 0.1798	− 0.2863	0.6607	12.2	99.2

The regression equation was statistically calculated according to correlation relationships between the photosynthetic parameters (the *X*_1,_ *X*_2,_ *X*_3,_ *X*_4,_ *X*_5_) and the silique *P*_n_ (*Y*) of rape variety, with *R* = 0.8529, *R*^2^ = 0.7275, and *P* = 0.0012, such as following:

$$Y = {6}.{9157}\,+\,{5}.{2}0{22}X_{{1}}\,-\,0.{1713}X_{{2}}\,+\,{16}.{3}0{6}0{5}X_{{3}}\,+\,{29}.{6423}X_{{4}}\,-\,0.00{34}X_{{5}} .$$

According to the statistical results, in influencing the silique *P*_n_ the first principal factors included the silique *G*_s_, Chl *a* and Chl *b* contents in silique hull, with contribution rate 65.5%; the second principal factor was the RuBisCO content in silique hull, with contribution rate 24.5%; the third principal factor was the silique *T*r, with contribution rate 6.5%. The cumulative contribution rate of these principal factors was about 96.5% (Table 6). Thus, the *G*s and *Chl a* and *Chl b* content of silique hull were the first principal factors that affected silique *P*_n._

**Table 6:** The principal factors which affected rapeseed silique *P*_n_.

Principal factor	Vector eigenvector of the variable parameter					Contribution percentage (%)	Cumulative contribution percentage (%)
	Silique *G*_s_ (*X*_1_)	Silique *T*_r_ (*X*_2_)	Chl *a* (*X*_3_)	Chl* b* (*X*_4_)	RuBisCO (*X*_5_)		
z1	0.5219	0.4439	0.4884	0.5259	0.1244	65.5	65.5
z2	0.0137	0.3687	− 0.3298	− 0.2175	0.8413	24.5	90.0
z3	0.1281	− 0.7498	0.424	− 0.0043	0.4916	6.5	96.5

### Effects of the shading experiment on the photosynthesis of leaf and silique and the seeds yield of rapeseed plant

Compared with the Control, under the ZH1 treatment the leaf *P*_n_, leaves area per plant, and siliques surface area per plant were significantly decreased, the seeds number per silique, and the siliques number, biological yield, seeds yield per plant was decreased by 30.9%, 39.0%, 21.3%, and 34.6%, respectively; while the silique *P*_n_ increased significantly, the 1000-seed weight was increased by 15.1%. Under the ZH2 treatment, the leaf *P*_n_, silique *P*_n_, and the leaves area, siliques surface area per plant were significantly decreased; the seeds number per silique, 1000-seed weight, and the siliques number, biological yield, seeds yield per plant was decreased by 32.3%, 39.0%, 74.3%, 68.5%, and 86.1%, respectively. The ZH3 treatment had no significant effects on leaf *P*_n_ and leaves area per plant, while the silique *P*_n_ and siliques surface area per plant were decreased significantly, ultimately the seeds number per silique, 1000-seed weight, and the siliques number, biological yield, seed yield per plant was decreased by 32.3%, 54.8%, 53.6%, 44.2%, and 84.3%, respectively (Table 7). In a conclusion, inhibition of silique photosynthesis of rapeseed plant during reproductive period led decrease of about 50% of siliques number per plant, 84% of seeds yield per plant, and 40% of biological yield per plant; inhibiting leaf photosynthesis led decrease of about 35% of siliques number per plant, 30% of seeds yield, and 20% of biological yield per plant; photosynthates supply in rapeseed plant during time from initial flowering to silique ripening influenced about 65% of biological yield and about 86% of seeds yield per plant; compared to that of the rape leaf photosynthesis, the silique photosynthesis largely influenced biological yield and seeds yield; the higher *P*_n_ of silique hull markedly promoted 1000-seed weight.

**Table 7:** Influences of the shading treatments on photosynthetic parameters and yields of rapeseed plant.

Treatment	Leaf *P*_n_ (μmolCO_2_ m^−2^ s^−1^)	Leaves area per plant (m^2^)	Silique *P*_n_ (μmolCO_2_ m^−2^ s^−1^)	Siliques area per plant (m^2^)	Seeds number per silique		1000-seed weight (g)		Siliques number per plant		Biological yield per plant		Seeds yield per plant (g)	
					Mean value	± (%)	Mean value	± (%)	Mean value	± (%)	Mean value	± (%)	Mean value	± (%)
ZH1	5.14 b	0.090 b	12.28 a	0.141 b	15.3 b	− 30.9	4.45 a	+ 15.1	150 b	− 39.0	38.9 b	− 21.3	9.41 b	− 34.6
ZH2	5.06 b	0.092 b	1.66 c	0.040 d	15.0 b	− 32.3	2.36 c	− 39.0	63 d	− 74.3	15.6 d	− 68.5	2.00 c	− 86.1
ZH3	19.69 a	0.162a	1.78 c	0.059 c	15.0 b	− 32.3	1.75 d	− 54.8	114 c	− 53.6	27.6 c	− 44.2	2.26 c	− 84.3
Control	20.41 a	0.158 a	10.26 b	0.231 a	22.2 a	0.00	3.87 b	0.0	245 a	0.0	49.5 a	0.0	14.40 a	0.0

The ZH1, ZH2 and ZH3 represented treatments that shading rapeseed plant during flowering stage, during time from initial flowering to seed ripening, or during time from flowering ending to seed ripening, respectively; control meant that the rapeseed plant was cultured under natural light condition. The ± (%) represented change rate of mean value of index under shading treatment relative to the Control. The same below.

### Effects of the shading treatments on seed quality, seed oil components, and gene expression of the key enzymes related to fatty acid synthesis

Compared to the Control, the oil content in seed and the oleic acid (C18:1), linoleic acid (C18:2), linolenic acid (C18:3) contents in seed oil showed no significant variation under the treatment ZH1, while the glucosinolate and protein contents were significantly increased. Under the ZH2 and ZH3 treatment, the oil content in seed and oleic acid (C18:1) content in seed oil were significantly decreased, while the linoleic acid (C18:2) and linolenic acid (C18:3) contents in seed oil, and the protein content in seed were significantly increased (Table 8). Thus, the leaf photosynthesis inhibition of rapeseed plant during flowering period had no significant effects on seed oil content and fatty acids content in seed oil. The silique *P*_n_ and siliques surface area (photosynthetic area) greatly influenced fatty acids synthesis; the silique photosynthesis inhibition resulted in decrease of the seed oil content, and the oleic acid (C18:1) content in seed oil was decreased while the poly-unsaturated fatty acids such as the linoleic acid (C18:2), inolenic acid (C18:3) contents were increased. Photosynthates deficiency in plant during reproductive period led increase of protein content in seed.

**Table 8:** Influence of the shading treatments on seed quality and gene expression of the key enzymes related to fatty acids synthesis.

Treatment	Content in seed			Content in seed oil			Gene expression level		
	Glucosinolate (umol g^−1^)	Protein (%)	Oil (%)	Oleic acid (C18:1) (%)	Linoleic acid (C18:2) (%)	Linolenic acid (C18:3) (%)	*ACCase*	*FAD2*	*FAD3*
ZH1	41.76 a	26.12 b	33.23 a	60.64 a	18.15 c	10.01 c	1.160 b	1.119 a	0.984 a
ZH2	32.87 b	26.61 ab	25.38 b	54.79 b	21.53 b	11.14 b	1.354 a	1.108 ab	0.810 b
ZH3	33.48 b	27.32 a	20.82 c	49.96 c	24.66 a	13.20 a	0.885 c	0.988 b	0.598 c
Control	35.34 b	24.60 c	34.29 a	60.58 a	18.99 c	10.29 c	1.000 bc	1.000 ab	1.000 a

Compared to the Control, gene expression levels of the *ACCase*, *FAD2* and *FAD3* in green seed weren’t significantly changed under the ZH1 treatment. However, under the ZH2 treatment the *ACCase* gene expression level was significantly enhanced while the *FAD3* gene expression was inhibited; under the ZH3 treatment the gene expression levels of the *ACCase*, *FAD2* and *FAD3* were slightly or significantly decreased (Table 8). Overall, in rapeseed plant the leaf photosynthesis inhibition during flowering stage had no significant effects on gene expression levels of the *ACCase*, *FAD2*, and *FAD3*, while the silique photosynthesis inhibition induced decrease/change of gene expression levels of the *ACCase*, *FAD2* and *FAD3* in green seed.

## Discussion

The improvement of rice grain yield was associated with enhancement of the LAI, leaf *P*_n_, root dry weight, and root oxidation activity^18^. By improving LAI and leaf *P*_n_ at flowering stage in the *Brassica napus* L., the seeds yield and oil yield were increased^19^. According to an equation model, the statistical results indicated that increase of grain yield was mainly depended on enhancement of leaf *P*_n_ and LAI in wheat (*Triticum aestivum* L.) and maize (*Zea mays* L.)^20^. Reduction of leaves area led lower grain yield in Oat (*Avena sativa* L.)^21^. In this study, the leaf LAI, silique SAI, siliques number per plant, and aboveground biological yield of rapeseed variety were statistically classified as the first principal factors which influenced seeds yield, the leaf *P*_n_ and silique *P*_n_ were the second principal factors.

Grain yield increase in maize parental lines was mainly attributed to the improved chloroplast structure, higher *P*_n_, higher *G*_s_, and stronger photosynthetic capacity after anthesis^22^. Improvement of ear leaf photosynthesis led enhancement of grain yield of winter wheat^23^. Slight shading after anthesis delayed leaf senescence and enhanced photosynthesis, thus higher grain yield was gained^24^. Recovery of tiller development and photosynthesis rapidly occurred when the shaded winter wheat was cultivated under natural light again, finally increasing grains number per tiller and smaller reduction of grain yield was achieved^25^. Inhibition of non-foliar organ photosynthesis led decline of seeds dry weight and quality^26–28^. The carbon dioxide assimilation in boll-leaf system (including main-stem leaf, sympodial leaf and non-leaf organ) was significantly linear-correlated with single boll weight of cotton (*Gossypium hirsutum* L.), the photosynthesis of non-foliar organ is important in increasing cotton yield^29,30^. Of oilseed rape (*Brassica napus* L.) the leaf was main photosynthesis organ during flowering stage, the siliques layer rapidly formed after anthesis and was mainly photosynthesis organ in seed growing and filling stage^6,7,9,31,32^. At present it was found that about 86% of seeds yield was mainly determined by photosynthesis of rapeseed plant in the stage from initial flowering until seed ripening, and 30% of the seeds yield was influenced by leaf photosynthesis of rapeseed plant during flowering stage, the silique photosynthesis after anthesis affected about 84% of seeds yield; the silique *P*_n_ and siliques area per plant largely influenced seeds yield, the higher silique *P*_n_ induced higher 1000-seed weight.

According to previous studies, total chlorophyll, Chl *a* and Chl *b* contents were positively correlated with *P*_n_ of flag leaf in rice and wheat varieties^33,34^. Chlorophylls content and pigment-protein complex level were enhanced in rice plant by transgenic strategy, and then, light harvesting efficiency via photosystem II was enhanced, the photosynthetic capacity of field-grown transgenic plant was improved, ultimately the vegetative biomass and grain yield were increased by 30–40%^35^. By foliar application with 6-BA and KCl liquid solution and reopening stomatal aperture in Kentucky bluegrass plants, it was demonstrated that the *P*_n_, *G*_s_, and *T*_r_ were positively correlated with the stomatal aperture^36^. In C_3_ plants the enzyme RuBisCO was a key isomerase in photosynthesis metabolism or photorespiration metabolism, photosynthetic activity of the RuBisCO was regulated by light, Mg^2+^ concentration, pH, NADPH, and RuBisCO activase. Moreover, the RuBisCO was an important storage protein and accounted for about 50% of soluble protein in plant^37,38^. In this study, the *G*_s_, Chl *a* content were the first principal factors which influenced the leaf *P*_n_ of rapeseed plant, the *G*_s_, Chl *a* and *b* contents were the first principal factors which influenced the silique *P*_n_, the RuBisCO protein content was the second principal factor which influenced the *P*_n_ of leaf and silique.

The photosynthetic product glucose was substrate in synthesizing fatty acids, the sugar transportation in seed coat dominated sugar concentration and regulated oil synthesis efficiency in seed of oilseed rape plants, higher photosynthetic capacity of silique hull accelerated oil accumulation in seed^39,40^, the gene expression of the transcription factor *WRINKLED*1 in oil synthesis pathway in seed was influenced by photosynthetic activity of silique hull, the seed oil content was significantly influenced by both activity of the RuBisCO enzyme and gene expression level of the RuBisCO subunit *BnRBCS1A* in silique hull^39^. For treatments of shading-silique the seeds yield and seed oil content of rapeseed variety were significantly decreased, the oleic acid and linolenic acid contents in seed oil were decreased, and the erucidic acid content was increased^8^. At present under the ZH2 treatment (shading rapeseed plants during time from initial flowering until silique ripening) or the ZH3 treatment (shading rapeseed plants after anthesis), the rape silique *P*_n_, siliques surface area per plant, seed oil content, and oleic acid (C18:1) content in oil was significantly decreased, while the linoleic acid (C18:2) and linolenic acid (C18:3) content in seed oil was increased. However, the ZH1 treatment (shading rapeseed plants during flowering stage) had no significant impact on seed oil content and fatty acids proportion. In a word, the leaf photosynthesis and leaves area per plant during flowering stage hardly influenced fatty acids synthesis and oil accumulation in seed, by siliques photosynthesis after anthesis the photosynthates was mainly produced and supplied to biosynthesize fatty acids in seed, the photosynthates deficiency in rapeseed plant after flowering induced decrease of seed oil content, decrease of oleic acid (C18:1) content and increase of polyunsaturated fatty acids content in seed oil.

Under stresses such as drought, pod removal and shade treatment the protein concentration was increased and oil concentration was decreased in soybean seed^41,42^. Lighting shade induced expression of proteins involved in photosynthetic metabolism and stress defense/detoxification^43^. Similarly, the present shading treatments induced increase of protein content in mature seed, although the seed oil content was decreased.

Gene expression of the ACCase was directly correlated with change of lipid content in culture of the chlorella (*Synechococcus* sp.)^44^. Specifically expression of the ACCase gene in seed induced increase of the seed oil content in transgenic *Brassica napus* L*.*^45^. The FAD2 catalyzed transformation from oleic acid to linoleic acid, through depression of the FAD2 gene expression in developing seed in oilseed rape, soybean, and peanut, ultimately the oleic acid content was increased and the linoleic acid content was reduced in mature seed^46–50^. The FAD3 gene encodes a rate-limiting enzyme in synthesizing α-linolenic acid, gene over-expression of the FAD3 induced α-linolenic acid content increase and linoleic acid content decrease in seed of the *Arabidopsis* and in rice bran oil^51,52^. Fatty acids biosynthesis and oil accumulation in crop seed occurred early in seed filling stage and went on until seed maturing, the oil was rapidly accumulated in seed at late stage of seed maturing^53^; through photosynthesis of green silique and green seed, the ATP/reductant and carbon source were supplied in synthesizing fatty acids, meanwhile the enzymes in fatty acids synthesis pathway expressed in green seed^54–56^. The gene expression of these enzymes related to fatty acid biosynthesis was regulated by multiple factors such as temperature, Light, and wounding, and was up regulated at onset of seed maturing^57,58^. In this study, it was found that the inhibition of leaf photosynthesis of rapeseed plant during flowering stage induced no significant effects on gene expression of the ACCase, FAD2 and FAD3 in green seed, while gene expression of these enzymes was restrained/changed for photosynthesis inhibition of silique and green seed, in the case, it was calculated that the fatty acids biosynthesis and oil accumulation in seed (in green seed or mature seed) would be influenced; while the fatty acids synthesis was regulated by multiple biotic and abiotic factors, under the shading stress, the variation of gene expression of the *ACCase*, *FAD2* and *FAD3* in green seed was not consistent with the changes of oil content and fatty acids proportion in mature seed. In sure, in seed filling and seed maturing stage the dynamic change of gene expression of these enzymes, and the dynamic accumulation of fatty acids in seed, as well as the relationship between gene expression of these enzymes and the fatty acids accumulation need a future research.

## Conclusion

In this study, the leaf LAI, silique SAI, siliques number per plant, and biological yield were statistically classified as the first principal factors which greatly influenced seeds yield of rapeseed variety (*Brassica napus* L.), the second principal factors involved the leaf *P*_n_ and silique *P*_n_. The *G*_s_ and Chl *a* content were the first principal factors which influenced leaf and silique *P*_n_ of rapeseed plant. For silique photosynthesis inhibition under shading stress, respectively the siliques number per plant, seeds number per silique, 1000-seed weight and mature seeds yield was decreased by 50%, 30%, 50% and 84%, higher silique *P*_n_ accelerated the 1000-seed weight, and the seed oil content and oleic acid (C18:1) content in oil was significantly decreased, while the seed protein content, and the linoleic acid (C18:2), linolenic acid (C18:3) contents in seed oil were increased; the changes of seed oil content and fatty acids content in oil were not consistent with variation of gene expression levels of the ACCase, FAD2, and FAD3 in green seed. However, under the leaf photosynthesis inhibition, the seeds yield, siliques number per plant, and seeds number per silique, each was decreased by about 30%, the seed oil content and fatty acids content in oil weren’t influenced, the seed protein content was increased. Thus, the silique photosynthesis after anthesis largely dominated seeds yield and quality in *Brassica napus* L.; a physiological indexes system including the leaf LAI, silique SAI, siliques number per plant, biological yield per plant, and the *P*_n_*, G*s, Chl *a* content of leaf and silique, was concluded to screen rapeseed variety with both higher light utilization efficiency and higher seed yield.

## Data Availability

The datasets used and/or analyzed during the current study was available from the corresponding author on reasonable request.
